# Recurrent Paralysis of Ocular Muscles Associated with Pain

**Published:** 1905-09

**Authors:** Alex. W. Stirling

**Affiliations:** M.D., C.M. (Edin.), D. P. H. (Lond.); Ophthalmologist and Laryngologist to the Presbyterian, Wesley Memorial, and Tabernacle Hospitals, Atlanta, Georgia


ATLANTA

Journal-Record of Medicine.

Successor to Atlanta Medical and Surgical Journal, Established 1855, and Southern Medical Record, Established 1870.

OWNED BY THE ATLANTA MEDICAL JOURNAL CO.

Published Monthly.

VOL. VII. SEPTEMBER, 1905. No. 6.

BERNARD WOLFF, M.D., EDITOR, Nos. 319–20 Prudential.

M. B. HUTCHINS, M.D., BUSINESS MANAGER, 1007-1008 Century Bldg.

J. N. LeCONTE, M.D., ASSOCIATE EDITOR.

### ORIGINAL COMMUNICATIONS.

### RECURRENT PARALYSIS OF OCULAR MUSCLES ASSOCIATED WITH PAIN.*

By ALEX. W. STIRLING, M.D., C.M., (EDIN.), D.P.H. (LOND.),

Ophthalmologist and Laryngologist to the Presbyterian, Wesley Memorial, and Tabernacle Hospitals,

ATLANTA, GEORGIA.

The following is a good example of a case, of which only some forty have up till now been reported:

When I first saw the patient, in February, 1902, he was twenty-eight years old. He then told me that ten years previously he had had a slight attack, lasting for only a few days, but it was not till seven years later that he experienced the full force of his trouble, which has consisted in recurrences of excruciating pain in the left eye and temple, followed by paralysis of the third nerve. I shall first describe his condition as I found it at his first visit. He had been partaking freely of acetanilid, codeine, and morphine for the relief of his suffering, and in consequence was pale and

*Read before the Georgia State Medical Association, April 21, 1905.

slightly cyanosed, with a very weak pulse. The attack had begun the night before with pain, which had come to a height in an hour or two. The diameter of his right pupil was now 2mm, that of his left 6.5mm. Vision of both right and left eyes was full, 6-5 without H. m. Left accommodation was paralyzed, and he required a plus 3 D lens to read at twelve inches. The movements of the globe were still normal, and there was no ptosis. Left T was normal, and the interior of the eye was healthy. I prescribed croton, chloral hydrate, and gelsemium, to be begun when the effects of the other drugs had diminished, and two leeches. I saw him again on the third day, and found that the prescription had been of no benefit, and that soon after his visit to me the external muscles had begun to be affected. He had now absolute ptosis, and, though the eye did not then diverge, he could turn it outward, but in no other direction beyond the middle line. No movement of the sup. oblique was observed. The pupil was fully dilated. The pain had somewhat lessened. I advised arsenic, quinine, and strychnine. When I next saw the patient, six months later, the left pupil was still too large, but acted fairly well to light and to convergence. There was no ptosis, and the ocular movements were normal. He had occasional pain, and he now tells me that on waking his vision was poor for near objects, and that then he used and still uses eserine, each application enabling him to read for two or three hours, and making the pupil as small as the other, or smaller. Without eserine he had required a plus 3 D lens. There was a slight but apparent exophthalmos. I saw him a month afterwards, in September, when an acute attack of pain was just beginning, and prescribed dionin, with, as I expected, no benefit. Between then and now he has had attacks of pain every two to four weeks, with slight paresis once, in December.

Looking back over the history of the affection, as it has been in his case, we find in many points a close similarity to the majority of the others which have been published. On neither the father's nor the mother's side was there any nervous affection, and among seven brothers and four sisters in only one case—the sister just older than himself, who has migraine every fortnight. It is not usual to find the family history so free from nervous cases. His own general health seemed to be good: no malaria, no rheumatism, no specific history or symptom, no albuminuria, no diabetes; knee-jerks, nose, throat, and ears normal. His only trouble is a distinct tendency to constipation and dyspepsia. Four years ago his neck was hurt by an elevator, without apparent subsequent bad results.

In considering the frequency of the attacks, it is necessary to separate the pain from the paralysis. Seven years divide the first from the second attack of pain, which occurred four and a half years ago, since which the intervals between the attacks of pain, lasting each four or five days, have varied from fourteen to thirty days, but he has had altogether only four onsets of paralysis. The first was in 1889, from which he recovered in two months, the second a year later, from which he took three months to get well. The next, the following year, continued for six months, and from the last attack, a year afterwards, in February, 1902, he recovered in three months. Besides these there was also a slight paresis in December. But in speaking of recovery, it is necessary to modify its meaning, because the pupil has never become normal in size or action since the first attack, and since the second the ciliary muscle has been more or less paralyzed, so that he can not read with the affected eye without eserine or a plus 3 D lens, the distant vision being normal. This continued ciliary paresis is the only instance of the kind which I have seen recorded, but there is a general tendency towards chronic paresis of the iris after a number of attacks. The aching, which is very painful, sharp, and continuous, last twenty-four to thirty-six hours before any paresis appears. This patient thinks paresis would always have appeared had it not been for large doses of acetanilid, the only drug, he says, which is of any use. He, unlike many cases, does not vomit, unless sometimes on purpose, in order to relieve the pain.

The sensation of the face has not been diminished, but a soreness remains long after the acute pain has disappeared, and indeed now seems to be permanent. There is nothing remarkable during or after these attacks in relation to the amount of urine passed, the patient's manner, the movements of the facial or lingual muscles, the secretion of tears or of saliva—all of which have been abnormal in some other cases recorded. As regards sex, the case is one of the minority.

The F V was not taken during the height of an attack, but in an interval taken by the hand it was full. No scotoma was shown for red or green. At no time was there pain on pressing the globe back into the orbit.

Recurrent paralysis of an ocular motor nerve associated with pain is well exemplified in this case. The third is the nerve most commonly affected, and sometimes only the branches to the external muscles, but the others, and even the seventh, are not immune, and sometimes all are attacked at the same time. The tendency generally is towards a diminished degree of recovery between attacks.

Concerning the etiology of the affection, though Charcot called it ophthalmic migraine, though it is frequently found in neurotic families, and though a large proportion of those who ultimately suffer from it, and many who never do, have a history of periodic headaches on the same side, yet the fact that the pain is so prolonged (it is said that the pain of migraine never lasts less than six nor more than twenty-four hours) the absence of visual phenomena, and the restriction of the site of the pain make it probable that the disorder is not true migraine. Besides the permanent paresis in the iris and in the ciliary muscle seems sufficient to put the theory of any functional disorder out of court.

If it is not functional where is the lesion? Not in the cortex, because if there the paralysis would have been conjugate. Drs. Holmes-Spicer and Ormerod, in their description of a case, in which "voluntary closure of the lids is complete, but in ordinary unconscious blinking the left (paralyzed) lids sometimes do not close at all," give this as an exemplification of those cases confirmatory of Mendel's hypothesis. Mendel's hypothesis, which has proven to be almost certainly correct, is that the orbicularis muscle is supplied by the nucleus of the third nerve, though the fibers run in the seventh nerve. But the fibers from the third nerve to the seventh enter the latter above its nucleus, because lesions of the seventh at the level of the nucleus or below paralyse the orbicularis. It follows that if one and the same lesion in their case paralyzed the third nerve and the orbicularis, it must have been a cortical lesion, and yet the paresis was not conjugate, but one-sided.

It is probably not nuclear, because acute nuclear paralysis of the third nerve is usually a symptom of serious progressive disease, is generally binocular, is apt to affect the levator palpebræ little or not at all, and is not likely to be associated with pain in the third nerve.

It is probably not fascicular, because one would expect along with the ocular paralysis, crossed hemiplegia. There remain only the nerves between their appearance on the cerebral base and the eye. If the lesion lay within the orbit, we should probably have other local symptoms, such as tenderness there, least on pressure or movement, and there was none. Besides, there would be a likelihood of involvement of the optic nerve, and in only one of the cases which I have seen so far published was there any such thing. That was a case of partial optic atrophy, which may have been an entirely separate matter. In a recorded case also of double ophthalmoplegia externa, with limitation of F V, the optic nerve may have been affected. But in the case now under consideration at least the paralysis is almost certainly of basal origin, and as only the superior branch of the fifth nerve is involved, the pain being confined to the distribution of that division, the lesion must lie between the Gasserian ganglion and the sphenoidal fissure. It may here be observed that in basal lesions of the fifth nerve the third nerve is generally paralyzed.

It may have been noticed that I have stated that, at a time when the third nerve in this case was already apparently paralyzed, the eye was still movable to and from the middle line outwards. Indeed the patient believes it never was fixed in divergence, and could always be moved somewhat outwards, though we may assume that the completeness of the ptosis interfered with his observation on this point, especially as we know that the sixth nerve of one side communicates in the brain with the third of the opposite side. A certain series of sixth-nerve cases should be interesting in this connection. Of these, I have myself seen some half dozen. In them the external rectus alone is paralyzed, or absent, and yet the eye at rest does not turn inwards, but lies when at rest in the middle line. I do not advance any theory to account for the absence of deviation in these cases. But some of Sherrington's investigations are interesting in connection with them. He showed that "the cortical area for the contraction of a given group of muscles coincides with the area for the inhibition of the group antagonistic to it." He divided the nerves to all the muscles of the left eyeball of a monkey, except the external rectus, producing a condition resembling that of my patient. Stimulation of those cortical centers which cause a conjugate deviation of both eyes to the right resulted in a movement of the left eye toward the middle line. This is held to be due to a simultaneous inhibition of the opposing muscles, in this case the left external rectus. Other experiments show that "the cells, the activity of which is thus inhibited, must lie below the cerebral cortex."

In the case of this patient, he himself believed the foundation of his troubles lay in his digestive apparatus, and a toxæmia appears to the writer to be the most likely explanation. But why the paresis should be in part permanent is a question. It might, however, perhaps be answered by supposing a resulting destruction of nerve tissue, such as we frequently see in the optic nerve after an acute inflammation. Dr. Bates Block informs me that the patient came under his care on account of dyspepsia, and that he believes that the treatment directed towards the improvement of the condition of his abdominal organs has been of use in reducing the severity of the attacks of pain. Priestly Smith also mentions a case for which he prescribed with decided benefit regulation of diet and a pint of hot water night and morning.

In one case (recorded by Kljatschin) malaria appeared to be distinctly the cause, and large doses of quinine had a curative effect.

So far as I am aware, only four of the recorded cases have arrived at the post-mortem table. In one was found a fibro-chondroma of the third nerve. In the second were plastic exudations and fibrous adhesions, involving the coat of the third nerve. In the next were granulations containing tubercle bacilli around the third-nerve root, and in the fourth a small fibroma connected with the dura mater completely involved the third nerve.

*This other case* may be worth reporting, in a few words, though doubtfully belonging to the class under discussion. Betty J., a colored woman, forty-one years of age, nursing a baby seventeen months old, in July observed *swelling of the left lids.* Some days later severe pain began in and around the left eye and in the temple. A week or two later she consulted me concerning the pain, and also on account of diplopia, which was just beginning. I found then that all the branches of the third nerve acted normally. Four or five days later the left external rectus was paralyzed, with marked strabismus. Five days later the inferior rectus also showed slight though distinct paresis. The third nerve otherwise remained unaffected. The fundus, vision, knee-jerks and facial sensation were normal. There had been no vomiting and no constipation. Nothing was found in the orbits. No scotoma for colors. F V full. There was no polyuria.

There was no history of trauma or syphilis, but out of her twelve children four were born dead. I prescribed anti-specific drugs. The pain gradually diminished, and in thirty days from the beginning there was none, and the movements of the eye were normal.

Several cases of recurrent paresis of the sixth nerve are included in those gathered together by Drs. Holmes-Spicer and Ormerod,* and Drs. Wissering, Anderson and Ormerod have each recorded cases of one-sided ocular paralysis associated with œdema of the lids and pain.

Note.—This paper was written nearly two years ago, but not published because I desired to make further observations during an acute attack, which, however, has not occurred. I saw the patient the other day (on June 9, 1905) and his eye was still as described above.

---

* Trans. Ophth. Soc. of Great Britain, vol. xvi., p. 279.

